# Identification *Acroporidae* and *Favidae* by a newly approach called *Reef Identification Knowhow Application-Reconstructed by 3D Imagery* (RIKA-R3DI) Method

**DOI:** 10.1016/j.mex.2019.05.002

**Published:** 2019-05-07

**Authors:** Rika Kurniawan, Aprilia Ariestasari, Roiko Sunarwan Silalahi, Ita Karlina, Try Febrianto, Dedy Kurniawan, Viktor Amrifo, Muhammad Abrar, Agung Dhamar Syakti

**Affiliations:** a Marine Science and Fisheries Faculty – Raja Ali Haji Maritime University, Jl. Politeknik Senggarang-Tanjungpinang, Riau Islands Province, 29100, Indonesia; b Fisheries and Marine Science Faculty – Riau University, Kampus Bina Widya Km 12,5, Simpang Baru, Pekanbaru, 28293, Indonesia; c Research Center for Oceanography, Indonesian Institute of Sciences, Jl. Pasir Putih I Ancol Timur, 14430, Jakarta Utara, Indonesia; d Center for Maritime Biosciences Studies – Institute for Sciences and Community Service, Jenderal Soedirman University, Kampus Karangwangkal, Jl. dr. Suparno, Purwokerto, 53123, Indonesia

**Keywords:** *Identification Knowhow Application-Reconstructed by 3D Imagery* (RIKA-R3DI) Method, Coral identification, 3D imagery reconstruction, Coral reef, Coral finder, Acroporidae, Favidae

## Abstract

With an increase on coral reefs vulnerability worldwide, an efficient and an integrated monitoring technique are required. Photographic and video methods are now becoming more attractive rather than conventional technique to optimize diver time. In this research, the level of effectiveness of RIKA-R3DI method was evaluated using Agisoft Photoscan. The method was based on the reconstructed images in three dimensional image assisted by a computer with Agisoft Photoscan to facilitate data processing. This research aims to identify Acroporidae and Favidae as the models of coral reef-building species. The samples were collected from the waters of Beralas Pasir Island, Bintan Regency. Visual engineering was conducted in the form of 3D viewing with attention to the corallite shape and size, as well as the color of the corals. The result was successful in identifying 4 species, i.e. Acropora microphthalma, A. sarmentosa, Favia maritima, F. vietnamensis. The keys benefit of this methodologies are:

• The RIKA-R3DI method allows to reduce diving time during direct visual observation.
• The method has high accuracy and is non-invasive, since it does not touch directly to the coral.
• The application of RIKA-R3DI can be used to identified the coral species and evaluate the coral health status base on the percent coverage.

## Graphical abstract

**Figure:**
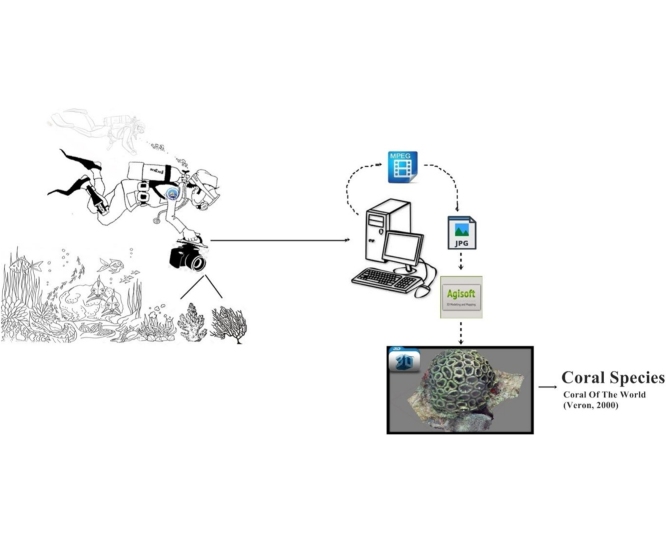

**Specifications Table**

**Table:** 

Subject Area:	Environmental Science
More specific subject area:	Coral ecology
Method name:	*Identification Knowhow Application-Reconstructed by 3D Imagery* (RIKA-R3DI) Method
Name and reference of original method:	• Methods for Ecological Monitoring of Coral Reefs [9] • Coral Finder [20].
Resource availability:	NA

## Method details

Indonesia is a maritime state with the longest coastline in the world, which has enormous potential in the field of marine and has approximately 95,000 km of coastline and a vast sea of around 5.8 million. This makes Indonesia a country that has the world's richest coral reefs, and it is estimated that there are more than 80 genera and 450 species of coral reefs in the region of Indonesia. Corals are widespread throughout Indonesia. The distribution of coral around the waters of Indonesia is uneven. The main factors that affect the vertical distribution are light intensity, brightness, temperature, and oxygen concentration of the water [1]. According to Burke et al. [2], coral reef ecosystem is one of the most productive systems on Earth, and most of the rich biodiversity is facing increasingly great threats, including the arrest of exaggeration, coastal development, runoff from agriculture, and the cruise. Facing such a risk, a monitoring research is a must for better managing the coral reefs. Researchers create methods that can facilitate their research in identifying or knowing the condition of coral reefs within a region. The used survey methods also vary from the method of Manta Tow [[3], [4], [5]], the method of Line Intercept Transect (LIT) [[6], [7], [8]], the method of Point Intercept Transect (PIT) [9,10], the method of Belt Transect [11], and the method of Quadratic Transek [12]. The following methods are often used in identifying or knowing the condition of the coral reefs in the area.

Furthermore, many researchers of coral in the world started to implement a new method that can simplify the coral research for those who do not have the expertise to identify it in the sea directly. One of the methods that are applied at the moment is the method of UPT (Under Water Photo Transect) [1,13], which utilizes a software, CPCe (Coral Point Count with Excel extensions), for processing data [14,15]. The previous methods lies in the way the samples are taken and processed. In the previous methods, the samples are mostly being processed under water while in some lesser extent the identification process might further conducted in the laboratory. This present study aims to propose a newly method of identification of genera and simultaneously assess the coral percentage by a method called RIKA-R3DI (Reef Identification Application Knowhow-Reconstructed by 3D Imagery). The principle of this method works on retrieving the data directly in the field (the sea) by photographing or video recording and then processing in the laboratory so that the samples will be identified in easy way independently without Scuba’a equipment.

## Materials and methods

### Sampling and research methods

The sampling techniques used in this research followed the purposive strategy [[16], [17], [18]]. where the location and sample to be taken are already determined directly by the researchers. The method used in this research is a survey conducted with the methods of observation, measurement, and sampling directly in the field and then processing the captured video to identify he samples by using a computer. The technique used is the observation method of RIKA-R3DI (Reef Identification Application Knowhow-Reconstructed by 3D Imagery), in which the sample will be taken using the underwater video and can be further analyzed in the laboratory. The duration of the sample videos are ±2 min, and all sides of the reef are taken thoroughly.

### Materials

As for the tools and materials used in this research, it can be seen in Table 1.

**Table 1:** Tools and materials used in the study of usability.

Materials	Usability
Scuba Underwater camera	For diving For taking the data on coral reefs
Ruler Agisoft Photoscan *Software* Coral Finder and Coral of the world Video Converter	For measuring the corallite coral reefs For processing the data For identifying the genus of coral reefs For splitting the video into photo

### Data analysis

Data analysis techniques used in this method are the method of visual engineering [19]. Visual engineering is done in the form of 3D viewing with attention to corallite formation, corallite size, and color of the coral reefs. In the process, investigators use Coral Book Finder [20] and Coral of the World [21] as the guide to determine the species of coral reefs.

## Results and discussion

RIKA-R3DI-method is combining a modified UPT method [1,13] and in situ methods (direct). The principle of this method is the same as in the method that utilizes UPT software to identify coral reefs, but in this method, the acquired data was taken by underwater camera/video recorder. The coral object video was transformed into imagery objects and then was reconstructed by using Agisoft Photoscan software with a 3D approach.

From the results of a survey that has been conducted in the waters of Beralas Pasir island, researchers took 3 samples from the *Acroporidae* and *Favidae,* which would then be identified on the ground later. The third sample will be processed and analyzed so that the morphology formations facilitate researchers in identifying the name of the species of each genus.

Samples taken did not focus on such sampling points in knowing the condition of the coral reefs in general. Samples were taken and determined directly by the researchers in accordance with the research that would be done. Samples that have been found are then taken using an underwater camera. The process of sampling favidae was followed by the measurement of corallite directly in the field using a ruler or caliper, and the aroporidae was looked for the corallite form and the original color on corals.

### Sample processing stages

**Fig. 1:**
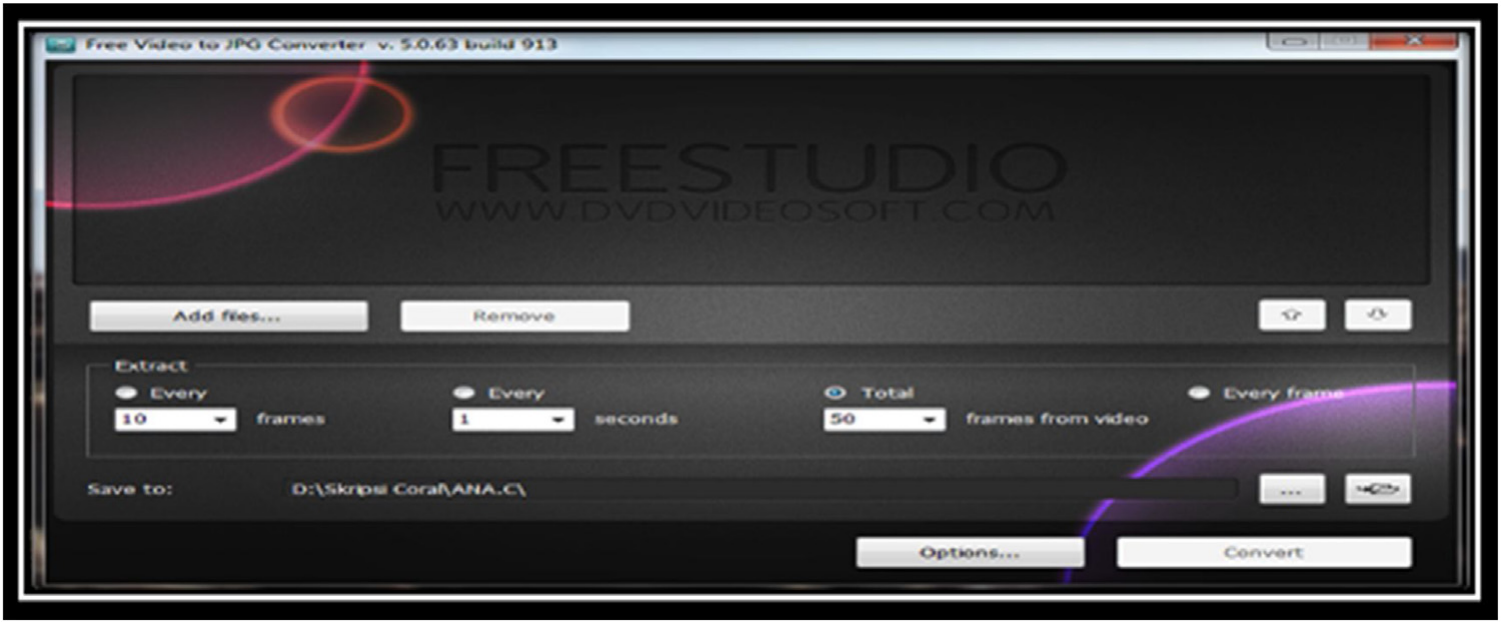
Display processing for video converter.
B Once the process is finished, the breakdown of the photos is cleaned using the photoshop software by pressing Ctrl + Shift + B (auto color) that serves to display the original color of those objects because, under identification, color plays an important role in determining species [22]. As for the look of the photoshop software, it can be seen in Fig. 2, Fig. 3.

Stages of processing, which are done in a sample of the video to convert it into a 3D display, are as follows:

A Video converter is used to make as many as 50 JPEG photos from the sample video. On the process of the study, the researchers split the video into 50 photos because the more the photos are broken into, the better it will be in getting good results for 3D viewing at a later stage. As for the look of the software, video converter can be seen in Fig. 1.

**Fig. 2:**
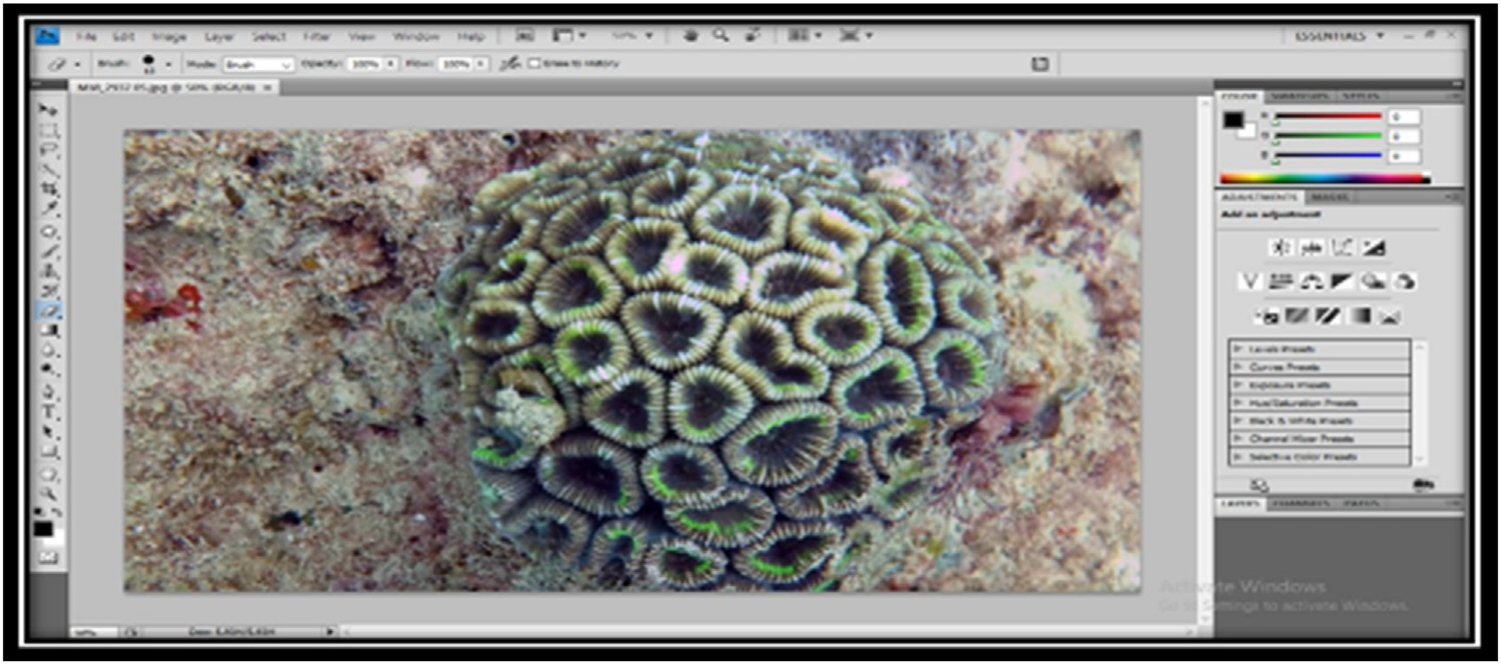
Display processing on photoshop software of Favidae.

**Fig. 3:**
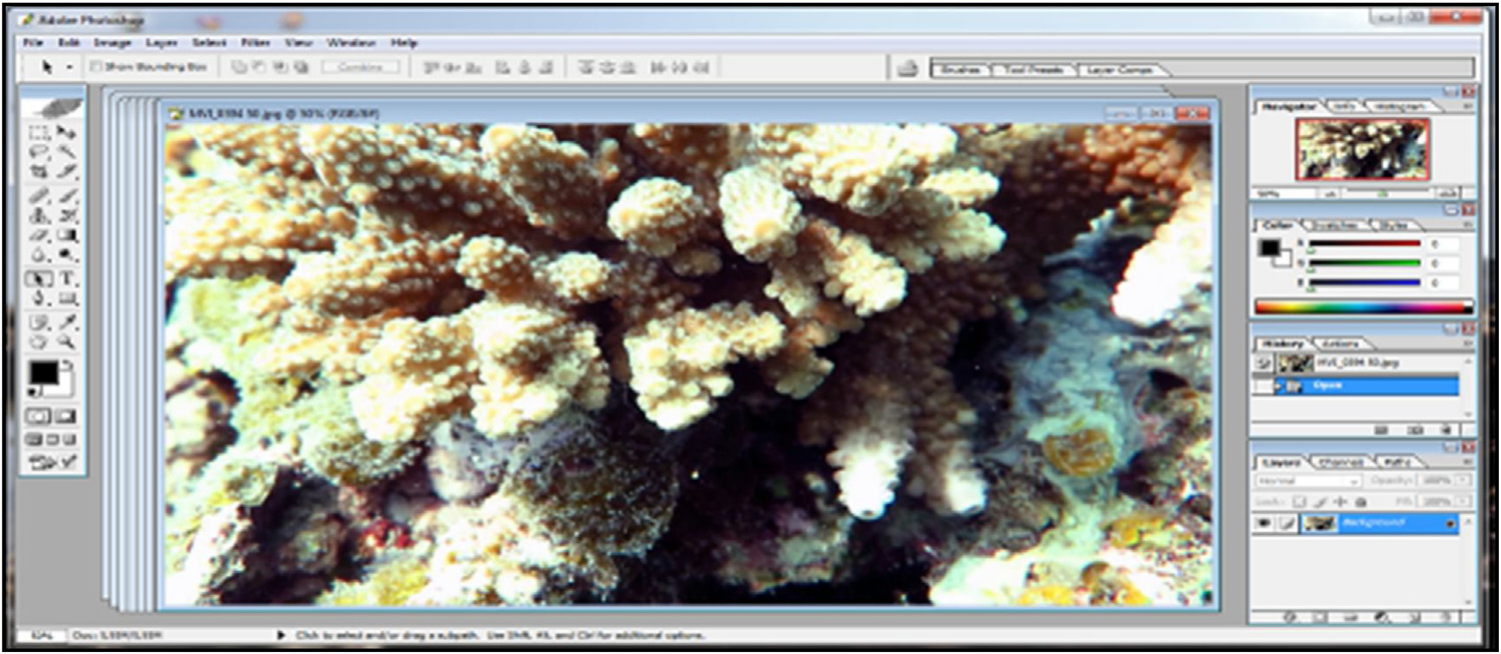
Display processing on software photoshop of Acroporidae.
C After the cleaning process, the color on the software is complete. The next sample that has been in the form of 50 photos were processed using the software Agisoft Photoscan back. The working principle of this software is to make a 3D display that can eventually facilitate researchers in identifying the sample. Data on corals that have been broken down into a number of photos are then processed into 3D form. As for some of the processes that must be performed to produce the perfect 3D shapes on the software, they are:
  1 Initial Process conducted in making a 3D display is the insertion of the photo data samples that have been broken down and wiped on the previous stage.
  2 The next step is to Align Photos on workflow tool, which functions to display and spruce up the photos so that the resulting 3D look neat and orderly.
  3 The next process is a pressing tool - Build Dense Cloud, which serves to thicken the result from the process - Align Photos.
  4 The next process is to Build the Mesh tool, which serves to clarify the results of the previous process. At this stage, the outcome of the resulting output has already started showing the form of a 3D sample but not completely perfect.
  5 The next process is to Build Texture tool, which serves to build and display the results of the 3D form that is more perfect. At this stage, the resulting 3D shapes are already showing the shape and color according to the original sample.
  6 The next process is a pressing tool - Build Tiled Model, which functions to produce a 3D form that is more clean and neat. At this stage, the produced 3D form is already much more perfect. The shape of the coral, the coral color, and shape of koralit are already showing similar results. Layout tools on software - Agisoft Photoscan are viewed in Fig. 4

**Fig. 4:**
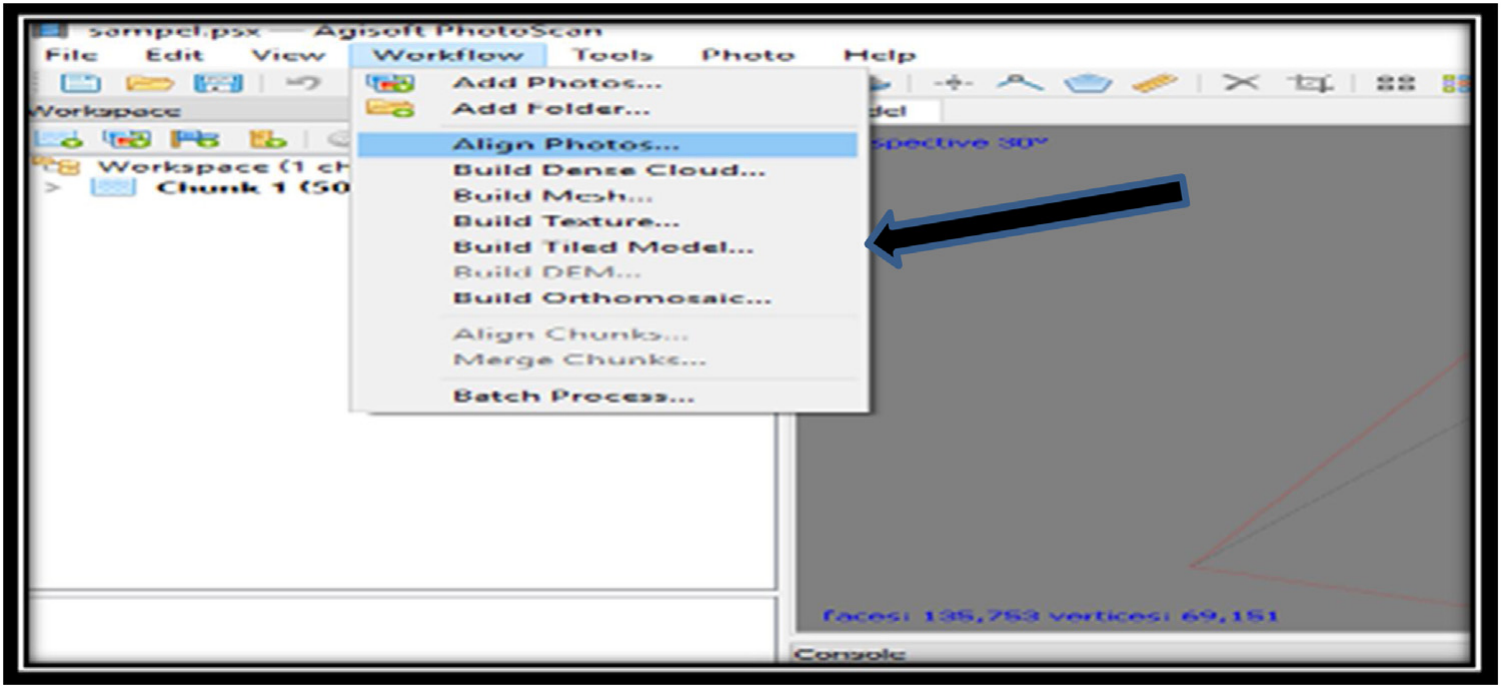
Layout tools on software Agisoft Photoscan.

**Fig. 5:**
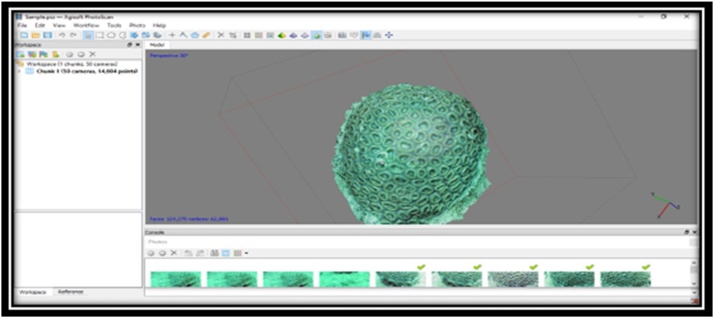
3D view on software – Agisoft Photoscan.

**Fig. 6:**
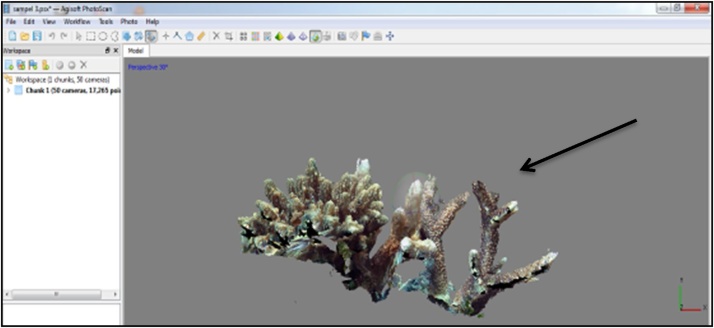
3D view on software – Agisoft Photoscan.

After the 3D form of coral appears, the new object can be identified visually with the help of the books, Coral Finder and Coral of the World, to know the genus and the species of the sample. As for the look of the software, Agisoft Photoscan can be viewed in Fig. 5, Fig. 6.

## The stages in identifying samples

**Fig. 7:**
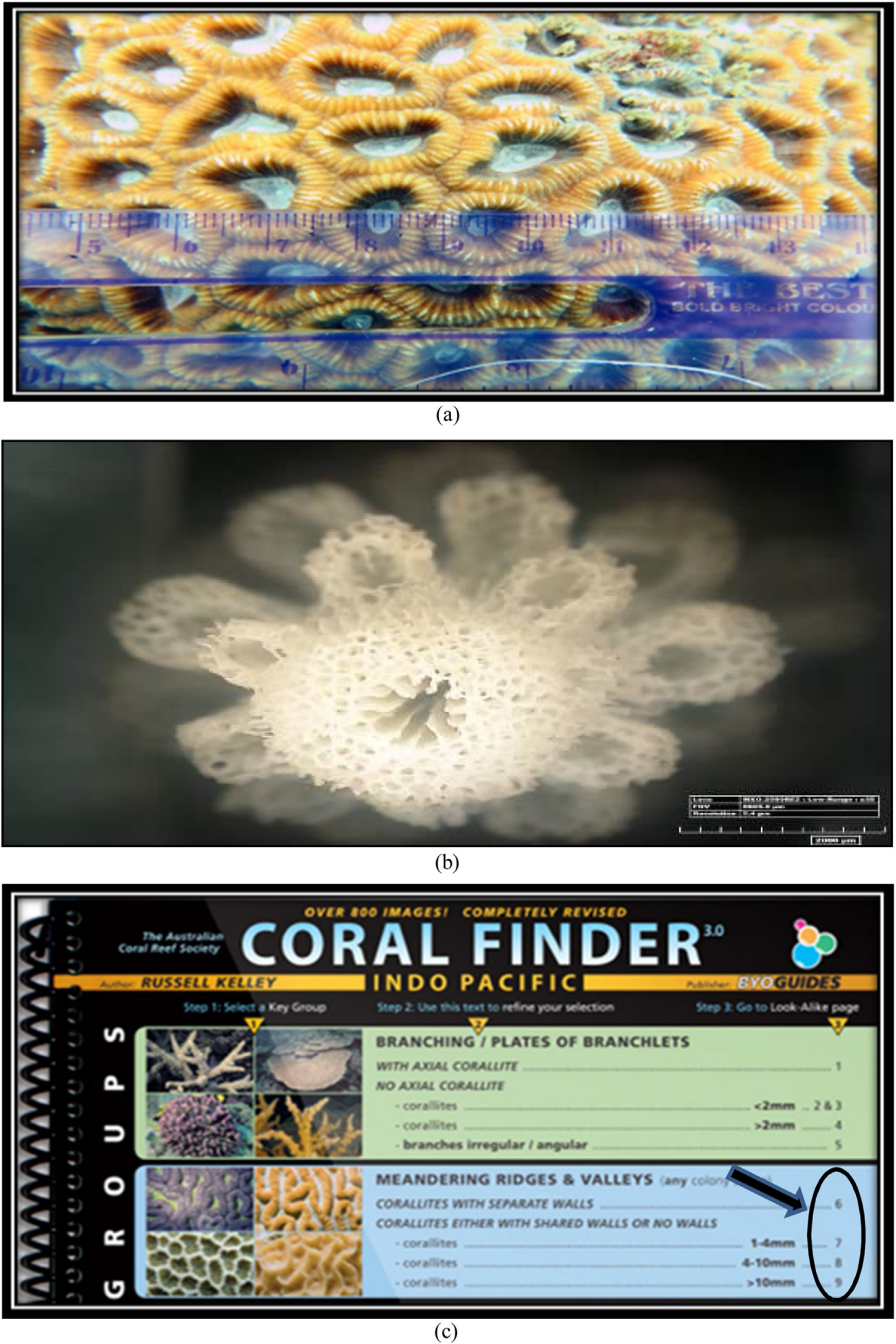
(a) the size of the corallite on the coral colony, (b) corallite formation of Acroporidae, and (c) coral corallite size determination by Coral Finder Method [20].
B After referring to a already directed view and after the koralit size of the corals have been known, next stage is to analyze the shape of the corallite and the color of the object to facilitate us in identifying the name of the genus of corals because the type and formation of the corallite on each genus corals will vary as it forms a dense corallite or separate, so it's important to know the shape of the corallite on the object in the stages of identification. Form corallite form analysis display and color objects can be seen in Fig. 8.

At the stage of identification, of course, we need to know the key and characteristics in identifying coral such as the size of the corallite, corallite formation, and the color of the coral colonies. The key and the characteristics stages that must be met in order for the species to be identified in accordance with the samples that were found. The following stages are performed in identifying the sample with a guide book - Coral Finder:

A The early stage in identifying the sample is done by paying attention to the morphological formations of the coral colony itself. The size of the coralliteis measured directly in the field, a key in the early stages of identification of genus of corals. After determining the large koralit, corals are identified, and then it will be instantly directed to a page where there are various types of coral with great corallite, already measured previously in the book - Coral Finder. The display size of corallite can be seen in Fig. 7.

**Fig. 8:**
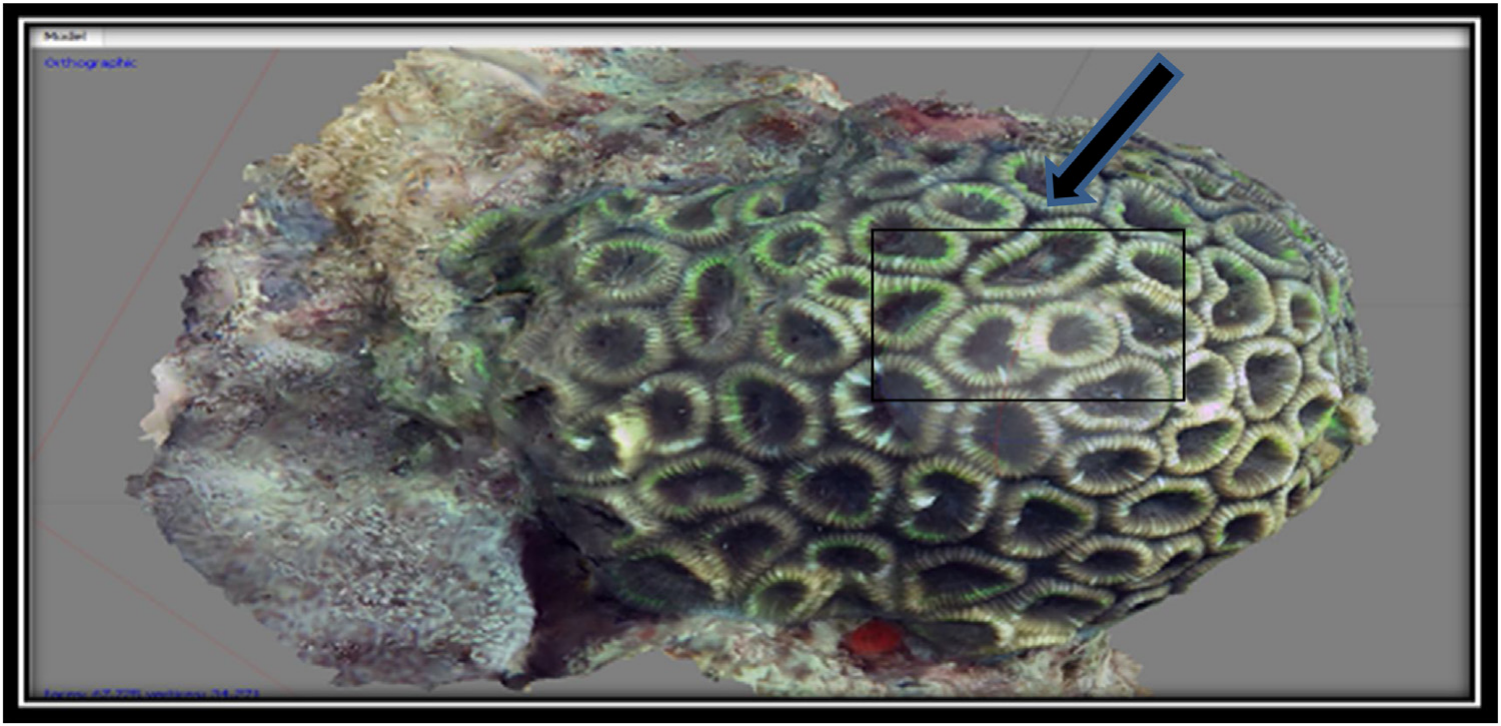
Display corallite shape analysis form and color on object.
C Once corallite size, corallite formation, and color of coral are already known, the next stage is the pursing option on the book, Coral Finder, to find out the name of a genus of objects that are characterized. We observe objects in accordance with the book - Coral Finder. Guide book - Coral Finder, for deciding the genus name of the object can be seen in Fig. 9. Samples can also were observed using a microscope Nikon Binoculars and Binoculars Optima Microscopes with 100–400 times of magnification.

**Fig. 9:**
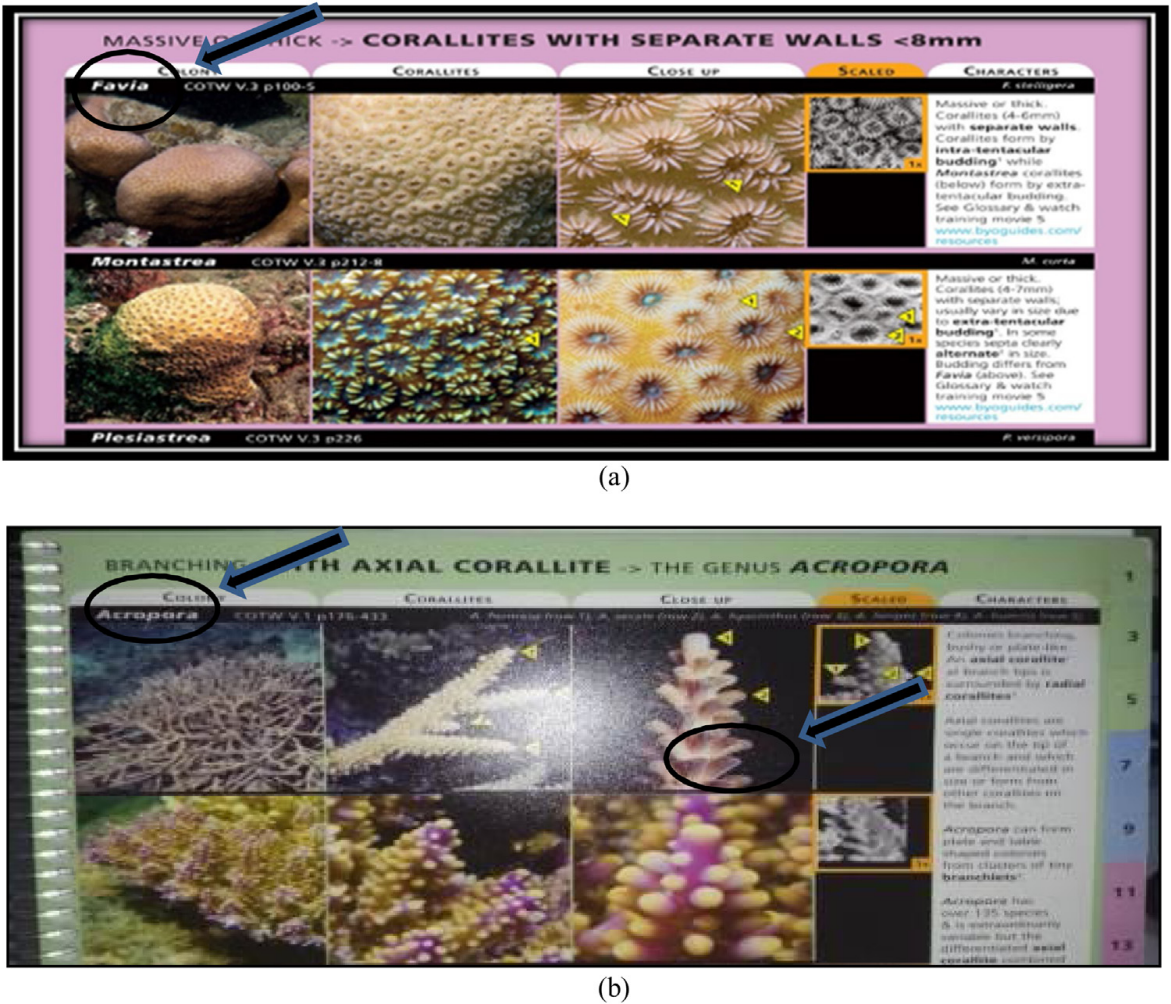
Guide book - Coral Finder, for deciding the genus.
D After the name of a genus of coral is known, the next stage is to determine the species name of the object. For specifying the name of coral species, researchers use a guide book - The Coral of the world. The specifying process should be done carefully because the shapes and colors of coral corallite can resemble other coral species. In determining the species of coral, the books, The Coral of the world and Coral Finder, already provide a Guide page that is referenced in the book - The Coral of the world so that the name of the species examined are valid. The guide reference page of the books and the book - Finder Coral Coral of the World can be seen in Fig. 10

**Fig. 10:**
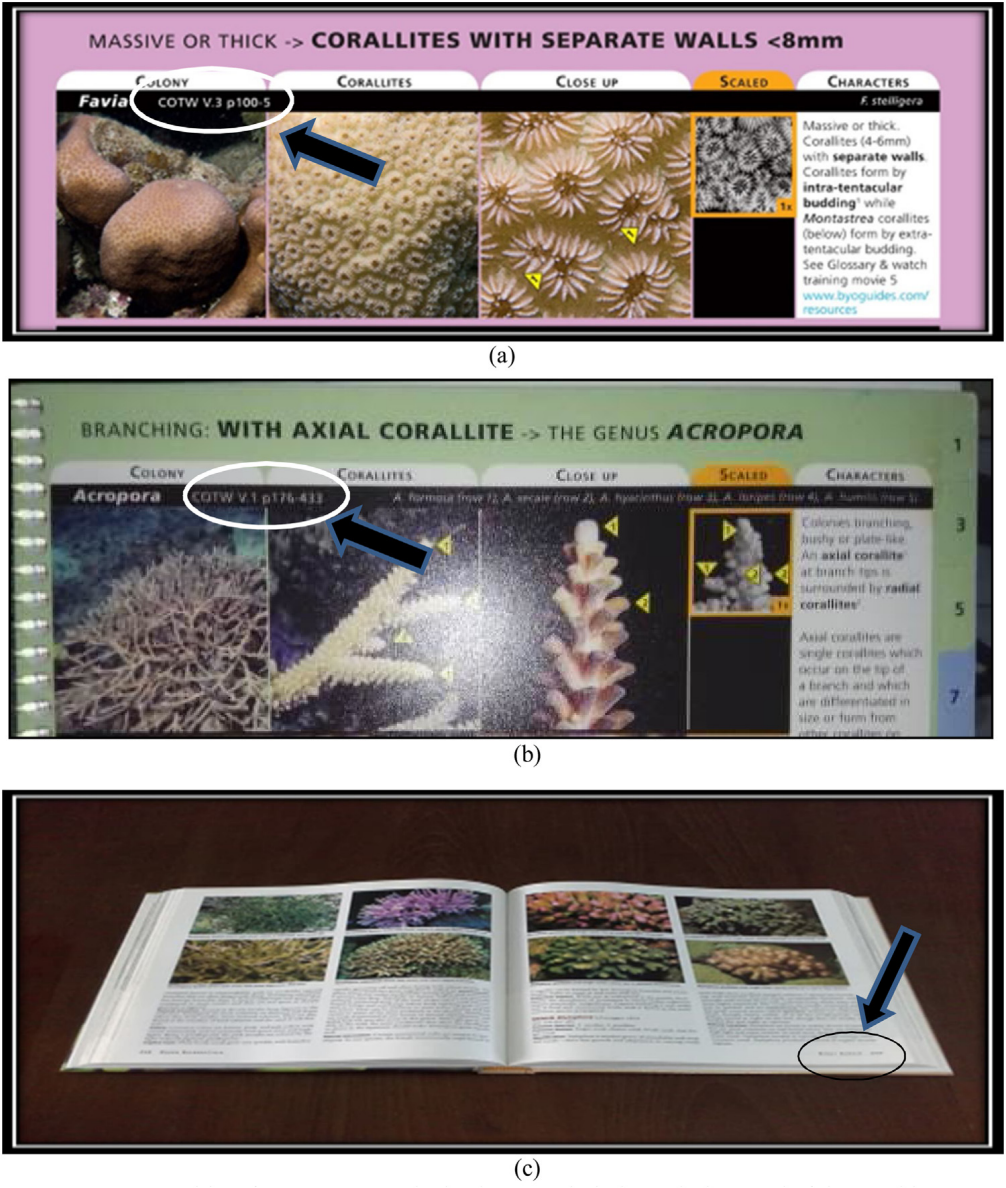
Guide reference page on the books - Coral Finder and The Coral of the World.

## The results of the identification of coral reefs

Based on the identification results that have been made, researchers have found 3 species of the Family *Acroporidae* and *Favidae*, the species which have been identified are as follows:

a ***Acropora sarmentosa*** (Brook, 1892)

**Fig. 11:**
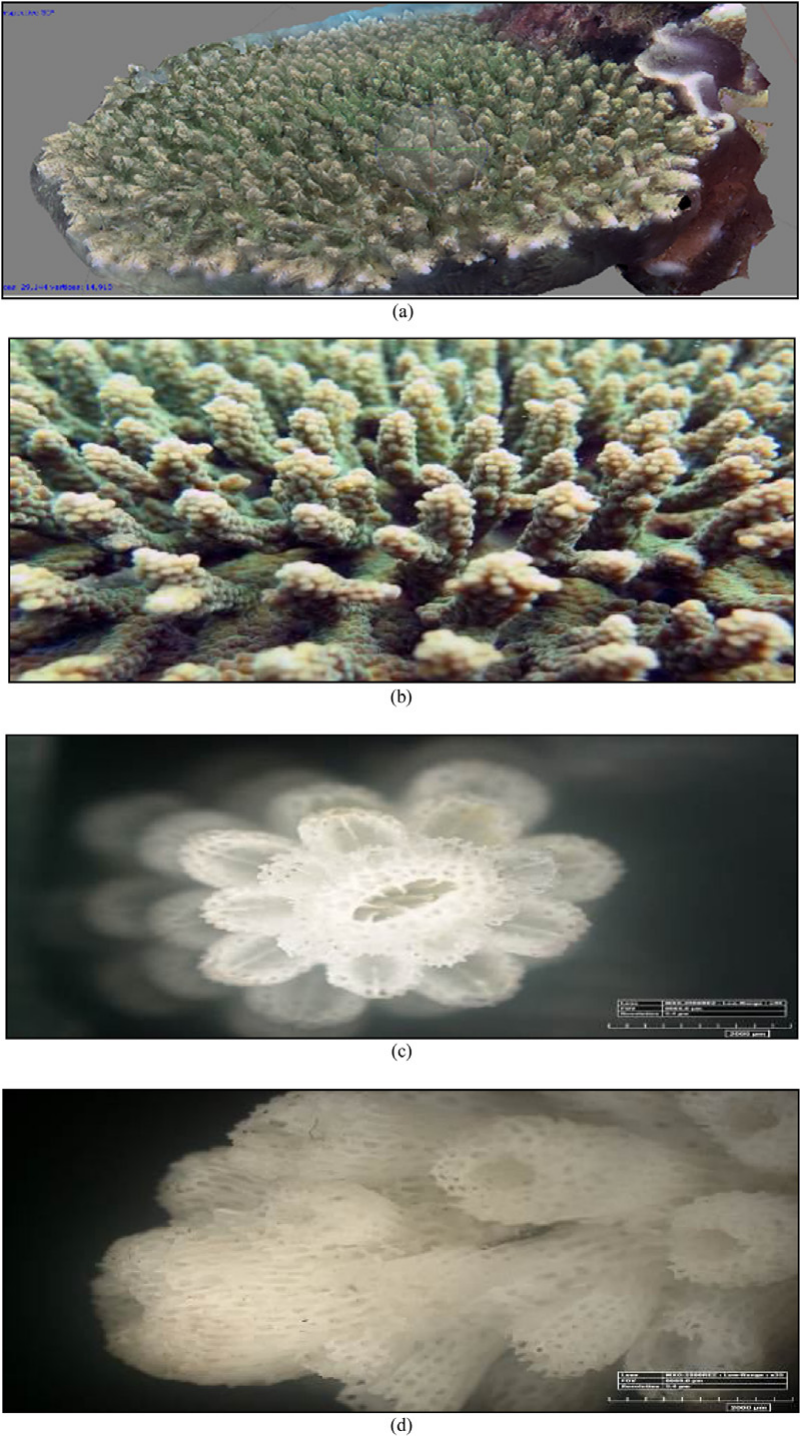
(a) The 3D form, (b) the original form, (c) and (d) the form of the koralit results. 35x magnification with a microscope of coral *A. sarmentosa* (Primary data, 2018).

The 3D form, the original form of reef, and the corallite formation results of 35x magnification of *A. Sarmentosa* with a microscope can be seen in Fig. 11 and Table 2.

• ***Acropora microphthalma*** (Verrill, 1859)

**Table 2:** The results of the identification of coral *A. sarmentosa* (Brook, 1892).

Genus	*Lifeform*	Corallite form	Colour	Species
Acropora	*Tabulate*	• Axial corallite big and round shaped. • Radial short size corallite with thick walls and blend with the axial corallite. *go to page 1 *Coral Finder*	Tanned green	Kingdom : Animalia Phylum : Cnidaria Class : Anthozoa Order : Scleractinia Family : Acroporidae Genus : Acropora Species : ***A. sarmentosa*** (Brook, 1892). COTW (*Corals of the World)* vol 1 p326.

Sumber: Coral finder [20] and Coral of the world vol 1 [21].

**Fig. 12:**
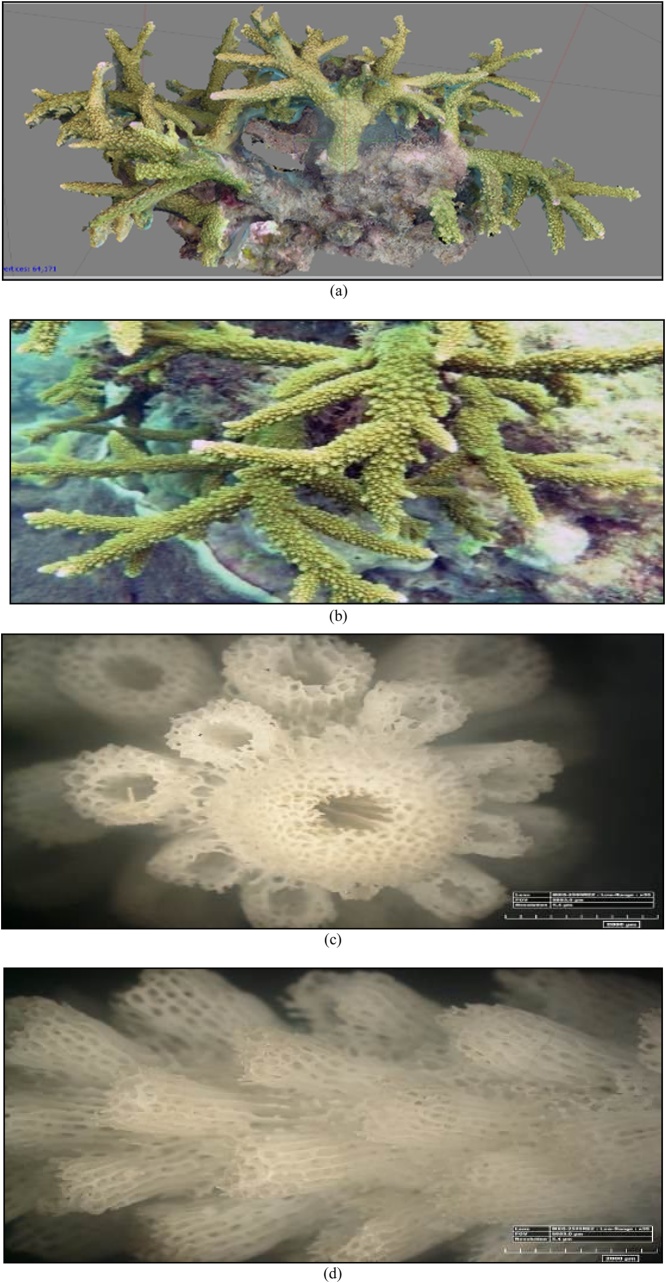
(a) The 3D form, (b) the original form, (c) and (d) the form of the koralit results. 35x magnification with a microscope of coral *A. microphthalma* (Primary data, 2018).

The 3D form, the original form of reef, and the corallite formation results of 35x magnification of *A. microphthalma* with a microscope can be seen in Fig. 12 and Table 3.

• ***Acropora microphthalma*** (Verrill, 1859)

**Table 3:** The results of the identification of coral *A. microphthalma* (Verrill, 1859).

Genus	*Lifeform*	Corallite form	Colour	Species
Acropora	*Branching*	• The radial corallite is small, many and the same size. *go to page 1 *Coral Finder*	Green brass yellow	Kingdom : Animalia Phylum : Cnidaria Class : Anthozoa Order : Scleractinia Family : Acroporidae Genus : Acropora Species : ***A. microphthalma*** (Brook, 1892). COTW (*Corals of the World)* vol 1 p258.

Sumber: Coral finder [20] and Coral of the World vol 1 [21].

**Fig. 13:**
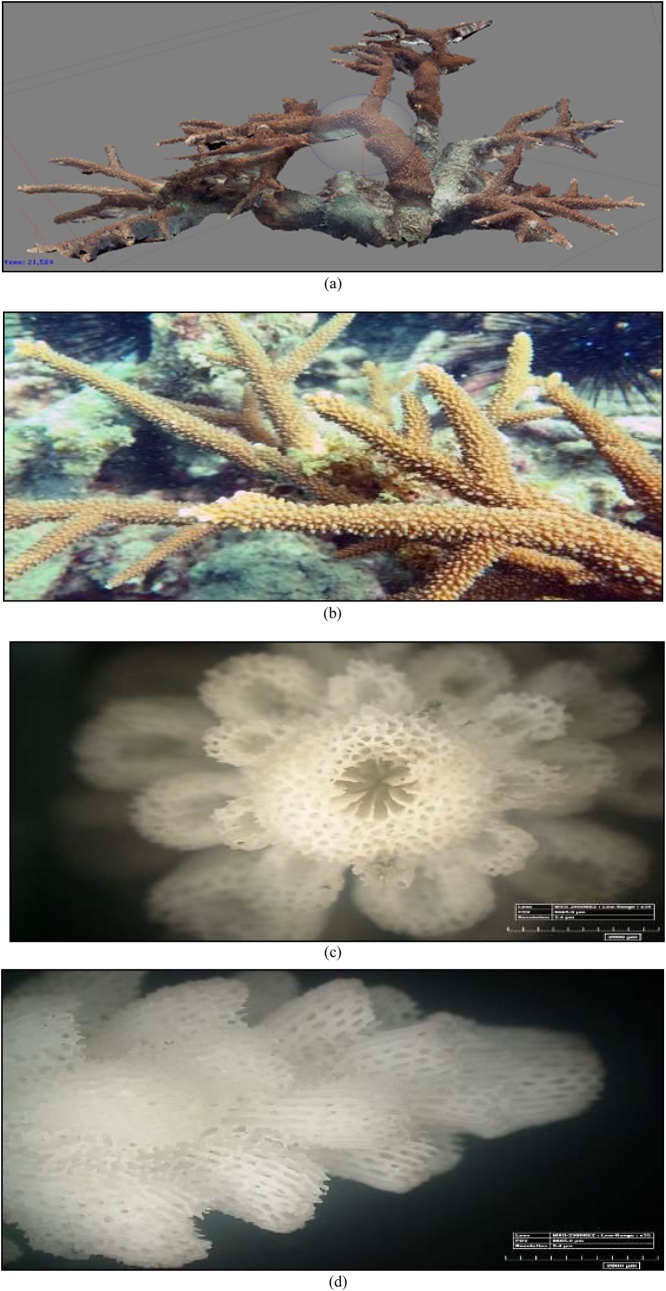
(a) The 3D form, (b) the original form, (c) and (d) the form of the koralit results. 35x magnification with a microscope of coral *A. microphthalma* (Primary data, 2018).

The 3D form, the original form of reef, and the corallite formation results of 35x magnification of *A. microphthalma* with a microscope can be seen in Fig. 13 and Table 4.

• ***Favia maritima*** (Nemenzo, 1971).

**Table 4:** The results of the identification of coral *A. microphthalma* (Verrill, 1859).

Genus	*Lifeform*	Corallite form	Colour	Species
Acropora	*Branching*	• The radial corallite is small, many and the same size. *go to page 1 *Coral Finder*	Brown	Kingdom : Animalia Phylum : Cnidaria Class : Anthozoa Order : Scleractinia Family : Acroporidae Genus : Acropora Species : *A. microphthalma* (Brook, 1892). COTW (*Corals of the World)* vol 1 p258.

Sumber: Coral finder [20] and Coral of the World vol 1 [21].

**Fig. 14:**
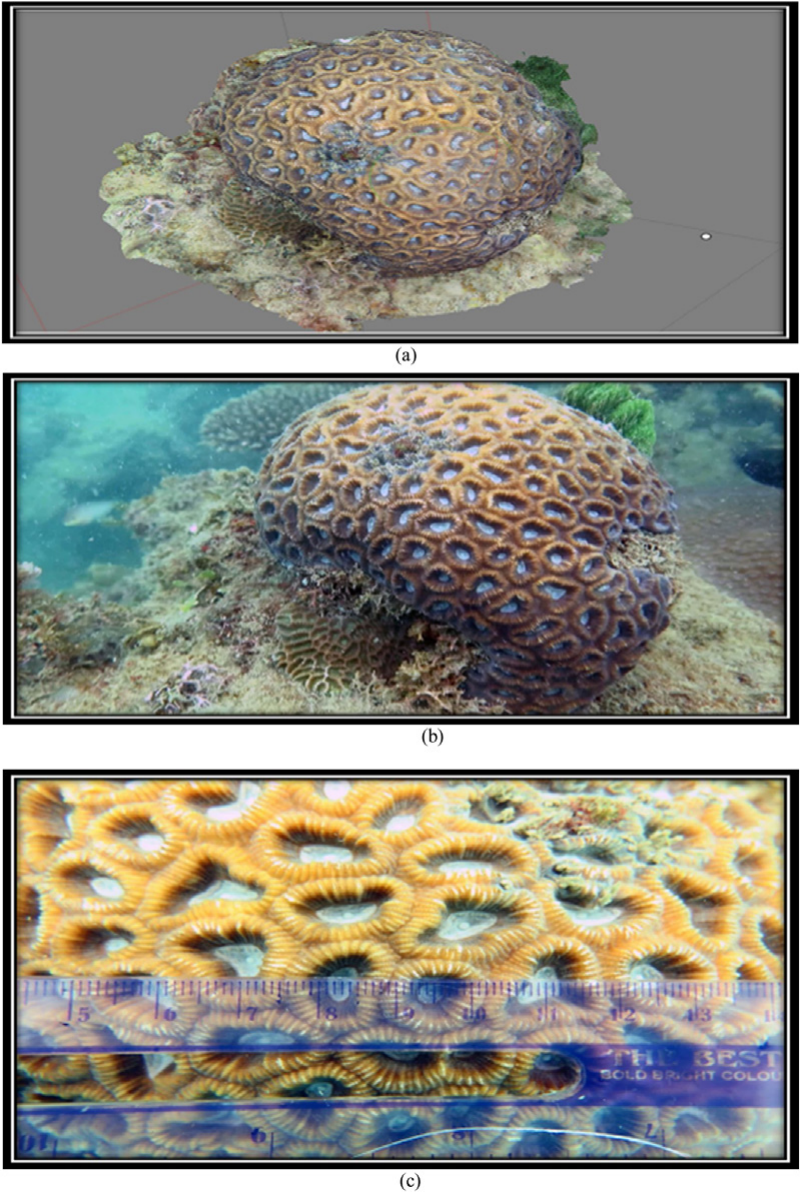
(a) 3D form, (b) original, and (c) corallite size of coral reef species *F.maritima* (Primary data, 2018).

The 3D form and the original form of species *F. Maritima* can be seen at Fig. 14 and Table 5.

• ***Favia maritima*** (Nemenzo, 1971)

**Table 5:** The results of the identification of coral *F.maritima* (Nemenzo, 1971).

Genus	*Lifeform*	Corallite form	Colour	Species
Favia	*Massive*	• Size of corallite 10-15 mm • Separate walls *go to page 11 *Coral Finder*	Dark brown	Kingdom : Animalia Phylum : Cnidaria Class : Anthozoa Order : Scleractinia Family : Favidae Genus : Favia Species : ***F.maritima*** (Nemenzo, 1971). COTW (*Corals of the World)* vol 3 p130.

Sumber: Coral finder [20] and Coral of the World vol 3 [21].

**Fig. 15:**
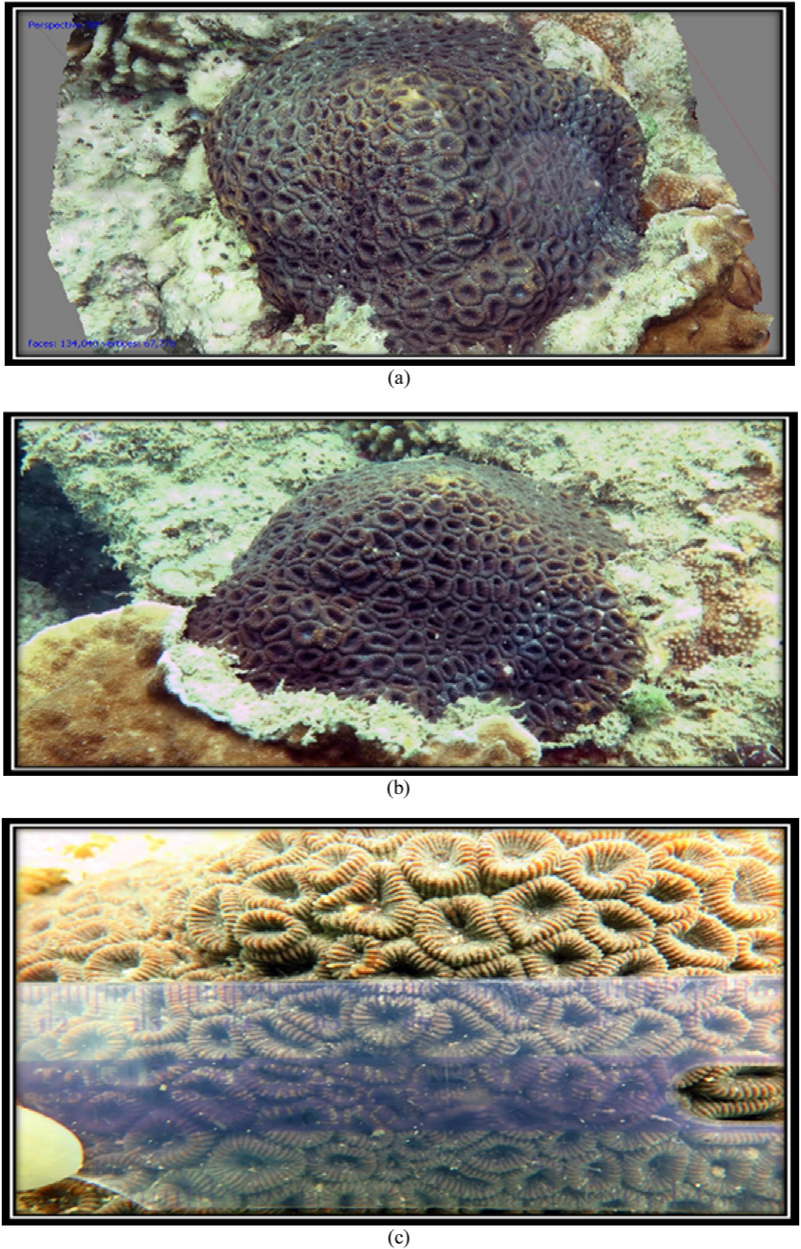
(a) 3D form, (b) original and (c) corallite size of coral reef species *F.maritima* (Primary data, 2018).

The 3D form and the original form of species *F. Maritima* can be seen at Fig. 15 and Table 6.

• ***Favia vietnamensis*** [21].

**Table 6:** The results of the identification of coral *F.maritima* (Nemenzo, 1971).

Genus	*Lifeform*	Corallite form	Colour	Species
Favia	*Massive*	• Size of corallite 9-13 mm • Separate walls *go to page 11 *Coral Finder*	Dark brown	Kingdom : Animalia Phylum : Cnidaria Class : Anthozoa Order : Scleractinia Family : Favidae Genus : Favia Species : ***F.maritima*** (Nemenzo, 1971). COTW (*Corals of the World)* vol 3 p130.

Sumber: Coral finder [20] and Coral of the World vol 3 [21].

**Fig. 16:**
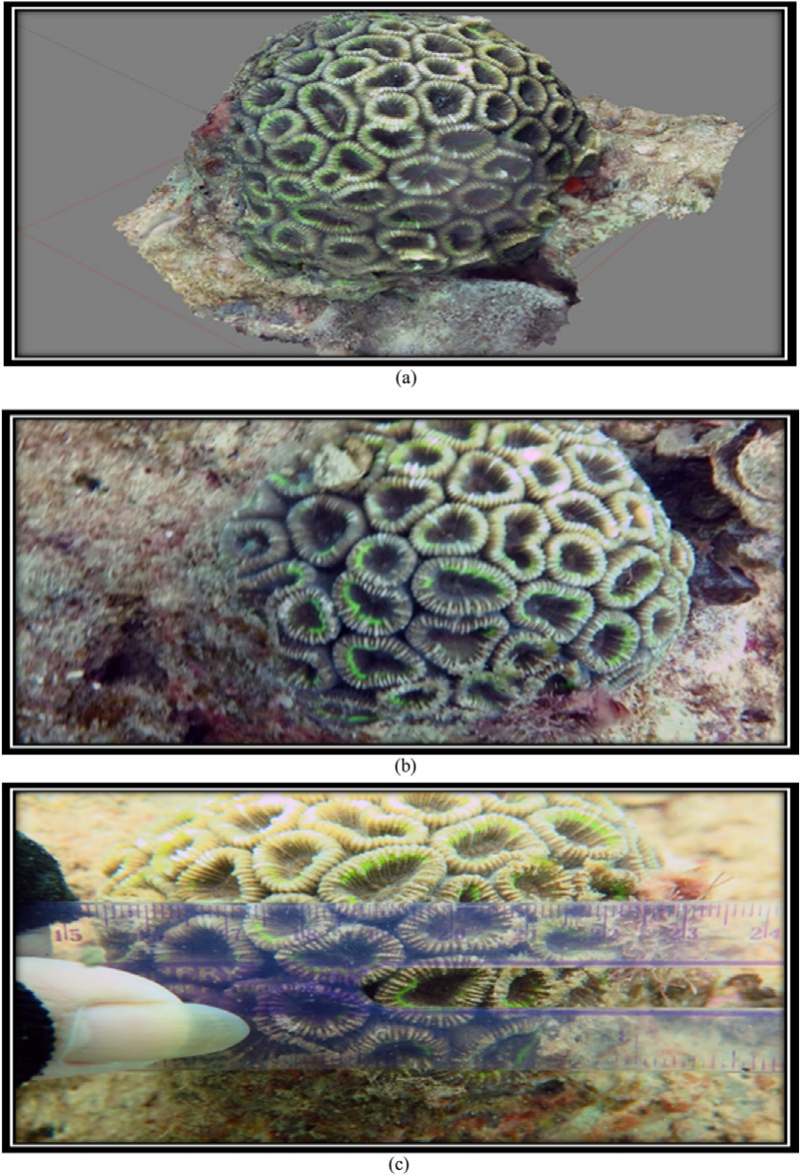
(a) 3D form, (b) original, and (c) corallite size of coral reef species *F.vietnamensis* (Primary data, 2018).

The 3D form and the original form of species *F. Maritima* can be seen at Fig. 16 and Table 7.

**Table 7:** The results of the identification of coral *F.vietnamensis* [21].

Genus	*Lifeform*	Corallite form	Colour	Species
Favia	*Massive*	• Size of corallite 11-14 mm • Separate walls *go to page 11 *Coral Finder*	Dark brown	Kingdom : Animalia Phylum : Cnidaria Class : Anthozoa Order : Scleractinia Family : Favidae Genus : Favia Species : *F. vietnamensis* [21]. COTW (*Corals of the World)* vol 3 p127.

Sumber: Coral finder [20] and Coral of the World vol 3 [21].

## Conclusion

The results of the research conducted in Beralas Pasir Island waters consisted of 6 samples that have been identified. The results of the identification using the books Coral Finder [20] and Coral of the World [21] consisted 4 species, i.e., *Acropora sarmentosa* (Brook, 1892), *A. microphlthama* (Verril, 1859), *Favia maritima* (Nemenzo 1971), and *F. vietnamensis* [21]. The results confirmed that the identification of *corals Identification Application Knowhow-Reconstructed by 3D Imagery* (RIKA-R3DI) method can be used in identifying the species of coral *Favidae* without having to bring the sample to the ground, while the *Acropridae* is still in need of treatment, where necessary sample is photographed in the field with a microscope so that the form of the corallites can be seen, and the identification of the corals is simplified for the researchers.

Coral colony structures generated in the form of 3D images through the Image Processing App (Agisoft Photoscan) have been much explored for identification tools but the identification process is still like conventional identification such as using coralite size with 2D images still possible. Some differentiating variable should be more elaborated, For instance, costae, sptae conesteum distance are not used for identification of the coralite wall of Favidae, event this information can be generated from the results of the Agisoft Photoscan Application. Future study will be advantageous for monitoring, especially when measuring the growth rate of coral colonies by measuring changes in component length, width and height in time series.
